# Variation in selected blood biochemical markers in green turtles (*Chelonia mydas*) from contrasting foraging habitats in Ecuador

**DOI:** 10.7717/peerj.21367

**Published:** 2026-07-29

**Authors:** Grace Johnston, Juan Pablo Muñoz-Pérez, Daniela Alarcón-Ruales, Felipe Vallejo, Cristina Miranda, Andrea Loyola, Gregory A. Lewbart

**Affiliations:** 1 The Department of Clinical Sciences, North Carolina State College of Veterinary Medicine, Raleigh, NC, United States of America; 2 Colegio de Ciencias Biológicas y Ambientales COCIBA, Universidad San Francisco de Quito USFQ, Quito, Ecuador; 3 School of Science, Technology, and Engineering, University of the Sunshine Coast, Sippy Downs, Australia; 4 Universidad San Francisco de Quito (USFQ) & UNC-Chapel Hill Galápagos Science Center (GSC), Isla San Cristobal, Galapagos, Ecuador; 5 Equilibrio Azul, Puerto Lopez, Ecuador; 6 Galápagos National Park, Puerto Ayora, Ecuador

**Keywords:** *Chelonia mydas*, Green turtle, Nutrition, Blood chemistry, Human interaction, Fisheries Impacts, Health

## Abstract

Green sea turtles (*Chelonia mydas)* are distributed throughout tropical and subtropical regions of the Atlantic, Pacific, and Indian oceans and are increasingly exposed to anthropogenic pressures in coastal aquatic habitats. The health impacts of anthropogenic pressures on green sea turtles remain unknown. This study evaluated biochemical differences between two neighboring Ecuadorian populations of Eastern Pacific green turtles to assess potential links between habitat use, diet, and physiological conditions and to discuss the causes of significant deviations in biochemical values. Blood samples were collected from 24 individuals and analyzed in situ using an iSTAT^®^ (Abbott Point-Of-Care) portable analyzer. Turtles sampled off the Ecuadorian mainland in Puerto López Bay, a site characterized by intensive commercial and recreational fishing activity, where turtles have been observed feeding on fishery discards, exhibited significantly increased levels of blood urea nitrogen (BUN) and blood glucose, and elevated total protein compared to turtles sampled in the protected Wreck Bay area of the Galápagos Islands, an area, where the turtles feed primarily on algae. Elevated biochemical values likely reflect altered dietary composition associated with anthropogenic food sources and increased protein intake. These findings highlight the influence of human-mediated foraging on green turtle physiology and provide information relevant to the management of green turtle health.

## Introduction

Green turtles (*Chelonia mydas)* are a keystone species in aquatic environments, playing an important role in structuring seagrass meadows and influencing nutrient cycling and ecosystem function (Bjorndal, 1980; Bjorndal, 1985; Thayer, Engel & Bjorndal, 1982; Johnson et al., 2020; Christianen et al., 2021). Despite their ecological importance, sea turtle populations are increasingly threatened by anthropogenic pressures, including marine traffic, poaching, environmental pollution, and entanglement in fishing gear and other debris (Parra Díaz, Deem & Espinoza, 2011; Nelms et al., 2016; Foley et al., 2019; Senko et al., 2022). The Eastern Pacific Distinct Population Segment of green turtles is currently listed as least concern by NOAA (Seminoff et al., 2015) and vulnerable (VU) on the *IUCN Red List of Threatened Species* in 2025 (IUCN, 2025).

Green sea turtles exhibit an ontogenetic dietary shift, transitioning from an omnivorous diet during juvenile stages to a primarily herbivorous diet in adulthood. Adult individuals predominantly consume seagrasses such as *Thalassia testudinum* and *Halophila* spp., along with various macroalgae, including *Ulva*, *Gracilaria*, and *Sargassum* (Bjorndal, 1980; Thayer, Engel & Bjorndal, 1982; Gulf Howell & Shaver). However, dietary composition can vary geographically and among populations. In Colombia, a green turtle population from Gorgona National Park presented a 63% frequency of invertebrate consumption (Amorocho & Reina, 2007), contrasting with populations in the Galápagos and Costa Rica, where turtles feed primarily on algae and aquatic vegetation (Carrión-Cortez, Zárate & Seminoff, 2010). Additionally, adult females may forage over broader spatial ranges and across multiple habitats compared to less mobile juveniles and males (Páez et al., 2022). Recent observations indicate that some individuals associate closely with small-scale fisheries and opportunistically feed on discarded fish remains, introducing an additional anthropogenic component to their foraging ecology. Such behavior has been documented in individual turtles through direct observation and stable isotope analyses in previous studies, suggesting that fisheries interactions can influence diet composition and trophic position (Turner Tomaszewicz et al., 2018). In this context, documenting behavioral evidence of scavenging and evaluating whether turtles foraging in human-impacted areas exhibit distinct physiological profiles may provide insight into the potential health implications of anthropogenic food subsidies (Clyde-Brockway et al., 2023).

Plant-based diets provide essential carbohydrates, vitamins (A, E, and C), and minerals such as calcium and magnesium, while maintaining a low-protein, high-fiber composition well suited to the slow metabolism and long lifespan of mature green turtles. However, the nutritional quality of these food sources can vary with environmental conditions; algae, in particular, may accumulate toxins or heavy metals in polluted habitats (Ankit, Korstad & Bauddh, 2022; Le et al., 2024). In areas where seagrass beds are degraded or absent, turtles may increasingly rely on algal resources, which may or may not meet their full nutritional requirements. Such dietary shifts can influence growth, reproduction, immune function, and disease susceptibility, including increased vulnerability to fibropapillomatosis (Duffy & Martindale, 2019; Howell & Shaver, 2021).

Baseline blood chemistry analysis is a widely used tool for assessing the health status and nutritional condition of green sea turtles in both wild and rehabilitative contexts. Biochemical parameters such as, glucose, total protein, cholesterol, electrolytes, and liver enzymes provide insights into metabolic function, hydration status, organ health, and nutritional balance (Flint et al., 2010; Lewbart et al., 2014; Kophamel et al., 2022; Stewart et al., 2023). Among these parameters, blood urea nitrogen (BUN) reflects protein metabolism and, although relatively insensitive, can provide information related to renal and hepatic function; elevated values may be associated with dehydration, gastrointestinal bleeding, shock, or high-protein diets (Salazar, 2014). Dehydration, gastrointestinal bleeding, shock, or a high-protein diet can lead to increased BUN in circulation. Blood glucose concentrations are also informative and may be elevated in chelonians due to dietary composition, stress responses, and gluconeogenic regulation (Stacy & Innis, 2017b).

Green turtle populations occur throughout Ecuadorian waters, including the continental coast and the Galápagos archipelago, where they represent the most common sea turtle species. Continental Ecuador and Galápagos populations are closely related and are observed year-round (Seminoff et al., 2008; Chaves et al., 2017). Puerto López Bay, located along the Ecuadorian mainland coast, is in a popular area of intense commercial and recreational fishing activity and is adjacent to a coastal town of approximately 12,600 inhabitants (Barragán Paladines, 2017; INEC, 2022). During fish processing, skeletal and visceral remains are commonly discarded into the ocean, where they are readily consumed by sea turtles and other scavengers such as crustaceans, marine birds and fish (Martínez-Ortiz et al., 2015; Barragán Paladines, 2017). In contrast, Wreck Bay in the Galápagos Islands is a protected foraging area associated with the town of Puerto Baquerizo Moreno (population ∼7,300; INEC, 2022), where green turtles primarily feed on natural algal resources.

This study evaluates differences in total protein, blood glucose, and blood urea nitrogen levels between green turtles from Puerto López Bay and Wreck Bay, to assess potential links between anthropogenic food sources, diet composition, and physiological condition. We hypothesized that green turtles foraging in areas with regular access to fisheries-derived food subsidies would exhibit elevated blood urea nitrogen, glucose, and total protein concentrations compared to turtles feeding primarily on natural algal resources in a protected area.

## Methods

### Study area

**Figure 1:**
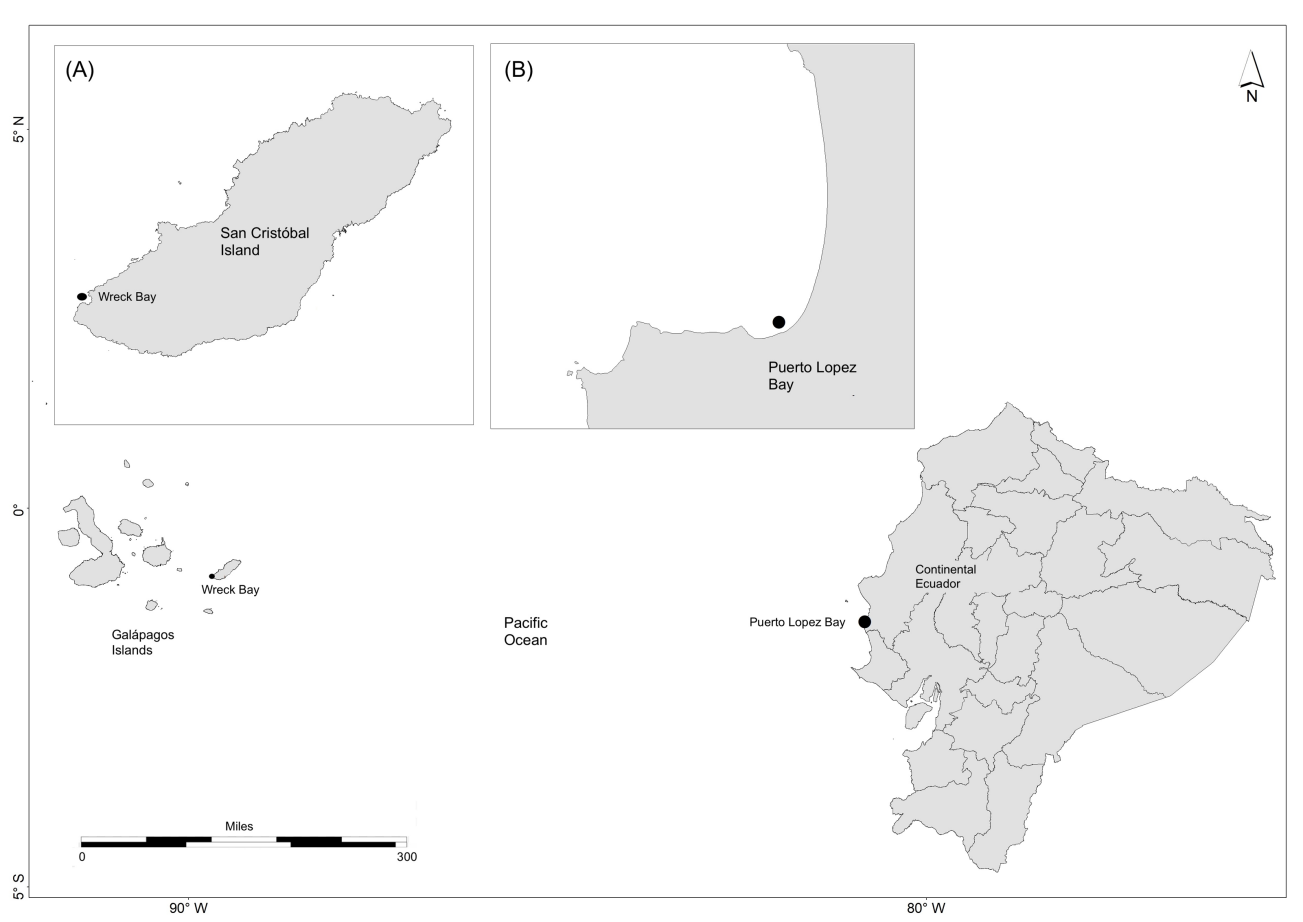
Map of the Galápagos Islands and Mainland Ecuador. Geographic distribution of sampling sites for juvenile *Chelonia mydas* in Ecuador. Locations include (A) Wreck Bay, San Cristóbal Island, Galápagos, Ecuador, and (B) Puerto López Bay, mainland Ecuador.

Sampling occurred at two coastal foraging sites in Ecuador that differ in protection status and human activity. Wreck Bay, San Cristóbal Island, Galápagos (−0.891366°, −89.616811°), is a protected embayment within the Galápagos Marine Reserve characterized by rocky substrates and abundant macroalgae, with minimal fishing activity. Puerto López Bay, mainland Ecuador (−1.559433°, −80.817638°), is a shallow coastal bay subject to frequent small-scale commercial and recreational fishing, where fish are commonly processed at sea and discarded remains are available to scavengers. The sites are separated by approximately 1,000 km. A map illustrating site locations and distances is provided in Fig. 1.

### Capture methods

Twenty-four juvenile to subadult green turtles (*Chelonia mydas*) were sampled using the methods outlined by Lewbart et al. (2014), Casale et al. (2016), Muñoz-Pérez et al. (2017). Turtles were encountered while foraging and were hand captured by swimmers equipped with snorkels, masks, and fins. Following capture, turtles were transported to a temporary onshore facility located within 100 m of the capture site for sampling.

To prevent resampling, a line of zinc oxide was put onto the carapace of the turtles prior to release. All sampled turtles were part of a larger study assessing microplastic ingestion in green turtles (Muñoz-Pérez et al., 2024).

### Ethical approvals and permits

Hand capturing and sampling were authorized by the Galápagos National Park Service (permit No. PC- 32-21 to J. P. Muñoz-Perez) and Machalilla National Park (permit No. MAE-EA-2021 to Felipe Vallejo). All handling and sampling procedures were consistent with standard vertebrate protocols and veterinary practices and approved by the North Carolina State University (IACUC Protocol #20-157).

### Blood collection and biochemical analysis

Turtles were manually restrained, and blood samples were collected from either the left or right dorsal jugular sinus using a three mL syringe with a heparinized 22-gauge needle. Blood samples were immediately divided into subsamples for instant blood chemistry measurements, hematological, and plasma analysis. The time from capture to blood sampling was 100.5 and 333.1 min, respectively, for Wreck Bay and Puerto López Bay. Blood biochemistry was analyzed *in situ* at both field sites using an iSTAT Portable Clinical Analyzer (Heska Corporation, Fort Collins, CO, USA) in both field sites. The iSTAT is a compact, portable, battery-operated electronic device with the ability to measure a wide variety of blood gas, chemistry, and basic hematology parameters with 0.095 mL of whole, non-coagulated blood. All biochemical analyses were performed using the CHEM8+ cartridges. Utilizing a portable microcentrifuge (Eppendorf North America, Inc., model 547), approximately 0.05 mL of blood was centrifuged for 5 min at 14,000 × g to determine the total protein (TP). TP values were recorded after one or two drops of plasma were placed in a refractometer (Advanced Optics, Oregon City, OR, USA).

### Statistical analysis

Mean values for blood urea nitrogen (BUN), blood glucose, and total protein were calculated separately for turtles sampled at Wreck Bay and Puerto López Bay. Statistical comparisons between populations were conducted using two-tailed *t*-tests assuming unequal variances. All calculations were performed using Microsoft Excel.

This study focused on glucose, BUN, and total protein due to their relevance to dietary protein intake and metabolic status.

### Behavioral verification of anthropogenic feeding

To verify scavenging behavior at Puerto López Bay, a Spatial Ecology and Scientific Video (SESV) device was deployed on one juvenile green sea turtle. The device was mounted at the cranial region of the carapace at the central dorsal level while the turtle was manually restrained. The SESV system, comprising a VEMCO VTP16 transmitter and a GoPro Hero3 camera, was secured to the carapace using a suction cup. The camera was oriented forward to capture the turtle’s field of view.

The SESV methodology integrates high-resolution active tracking telemetry with continuous video recording to examine marine turtle behavior and habitat utilization. This approach is adapted from Thomson & Heithaus (2014), employing a custom-designed animal-borne video recorder. The attachment procedure was performed in less than 10 min, minimizing handling time to reduce stress and prevent alterations in natural behaviour. This method was chosen because it provides fine-scale data while minimizing observer interference.

Following the release, approximately three hours of video footage were recorded. The footage documented repeated scavenging behavior, including the consumption of discarded fishery products and interactions among conspecifics competing for food resources. After the recording period, the turtle was relocated, manually restrained, and the SESV device was removed. The turtle was then released at the capture site.

## Results

**Figure 2:**
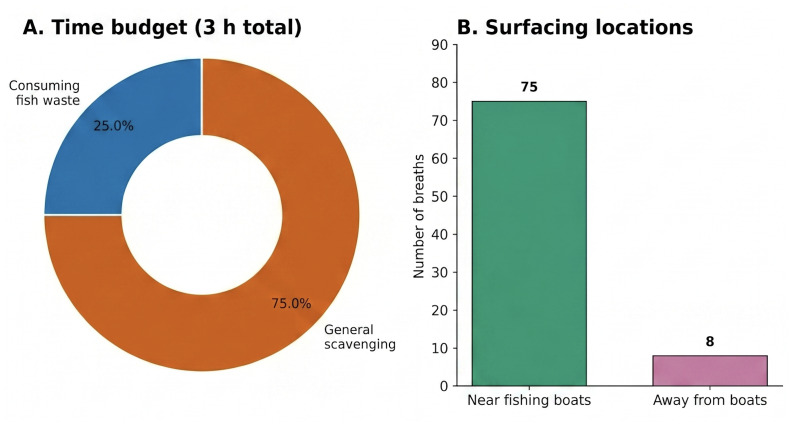
Sea turtle behavioral time budget. Behavioral time budget and surfacing locations of the observed sea turtle. (A) Time budget analysis showing the percentage of time spent consuming discarded fish waste *versus* general scavenging activities during three hours of continuous footage. The turtle dedicated 25% (45 min) of the observation period to consuming discards. (B) Frequency of surfacing events (breaths) recorded relative to the proximity of fishing vessels (*n* = 83 total breaths). The turtle surfaced near fishing boats for 75 breaths (90.4%), indicating high site fidelity to the fishing grounds.

Juvenile to sub-adult (curved carapace length, CCL, 44–70 cm) green sea turtles of both sexes were repeatedly observed scavenging on discarded fish carcasses in Puerto López Bay. Video footage collected over a three-hour deployment on a single turtle documented continuous scavenging behavior with no extended resting periods. The focal turtle was observed consuming or attempting to consume discarded fish waste for approximately 45 min of the total recording time and was recorded competing with conspecifics for access to fish remains (Fig. 2). The footage also documented turtles aggregating around anthropogenic structures, including anchor lines, ropes, and discarded polyvinyl chloride (PVC) pipes with attached debris.

**Figure 3:**
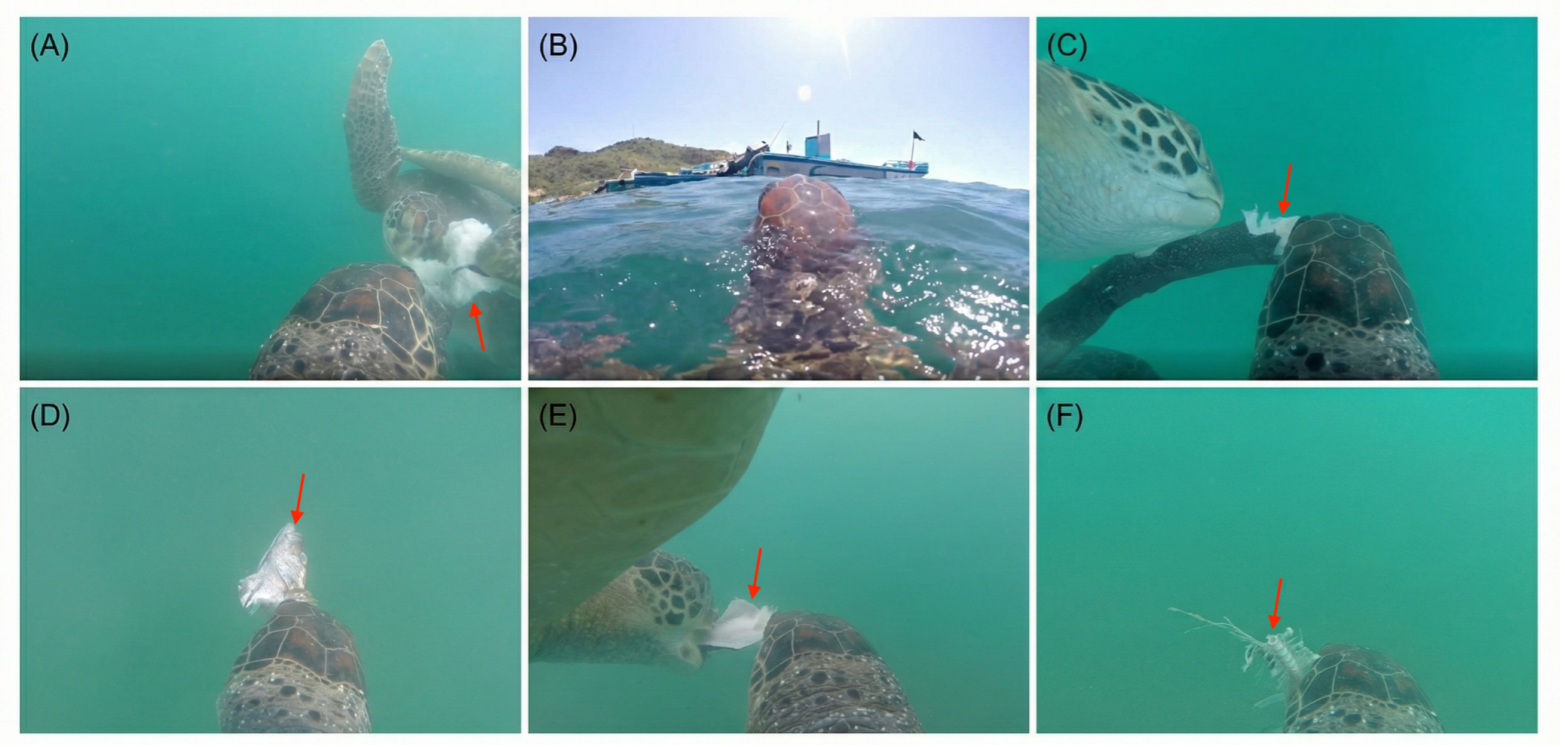
Images of scavenging green turtles. Examples of interactions and scavenging behavior of green sea turtles (*Chelonia mydas*) on discarded fish in Puerto López Bay, Ecuador. Frames were captured using a camera-mounted Spatial Ecology and Scientific Video (SESV) device. (A) Two conspecifics attempt to compete for the fish carcass being scavenged by the camera-tagged turtle. (B) A sea turtle surfaces to breathe near the camera-tagged individual, demonstrating the proximity of fishing vessels during scavenging. (C) Active scavenging behavior. (D) The camera-tagged turtle feeding on a discarded fish head. (E) Another conspecific entering the frame to contest the food source. (F) Additional perspective of the turtle active scavenging behavior.

Respiration data indicated that the tagged turtle remained within the fishing grounds throughout the deployment. Of the 83 surfacing events recorded, 75 occurred in close proximity to fishing vessels, indicating sustained association with fishing activity despite prior capture and handling for camera attachment (Figs. 2 and 3).

**Figure 4:**
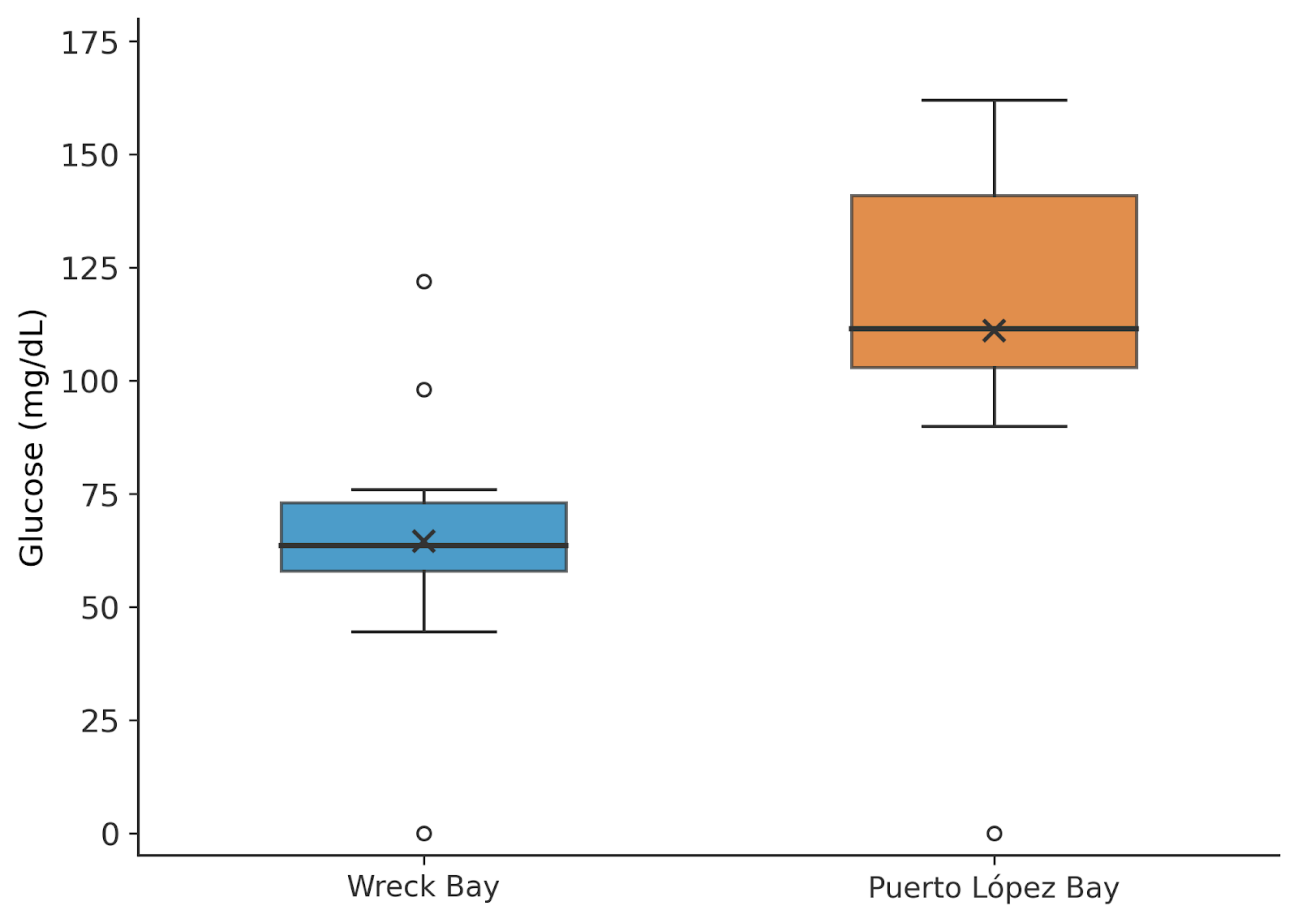
Blood glucose levels comparison. Elevated blood glucose concentrations in green sea turtles (*Chelonia mydas*) from Puerto López Bay compared to Wreck Bay. Mean blood glucose concentrations were significantly higher in the mainland coastal site (122.6 mg/dL) *versus* the insular Galápagos site (69.2 mg/dL) (*p* < 0.000009). Error bars indicate standard error of the mean.

**Figure 5:**
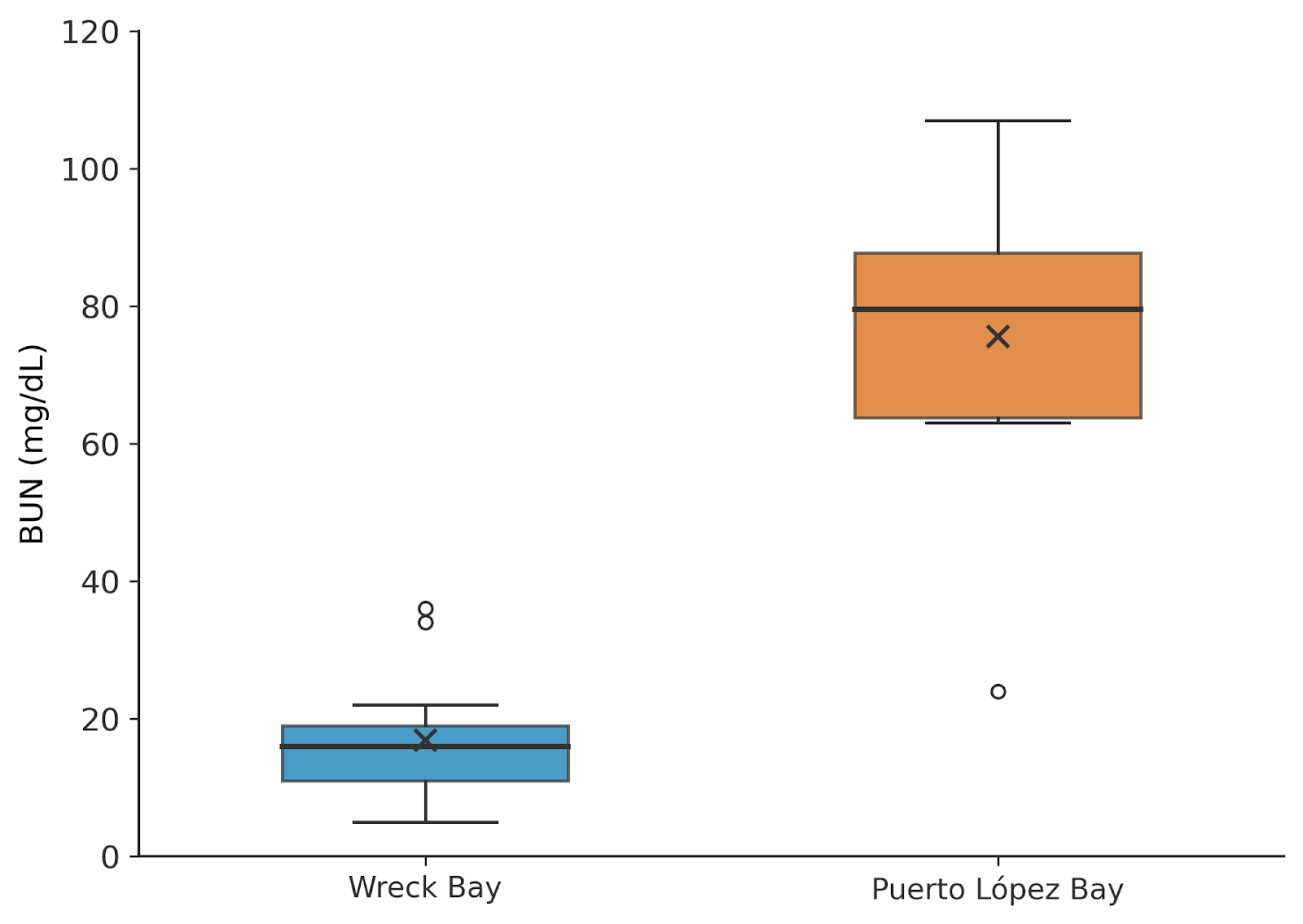
Blood urea nitrogen (BUN) levels comparison. Comparison of mean blood urea nitrogen (BUN) concentrations in green sea turtles (*Chelonia mydas*) between populations. Turtles sampled adjacent to the Ecuadorian mainland exhibited significantly higher BUN levels (Mean = 75.6 mg/dL) compared to the Galápagos population (Mean = 18.9 mg/dL). This elevation in the mainland group was statistically significant (*p* < 0.000024). Error bars represent standard deviation.

**Figure 6:**
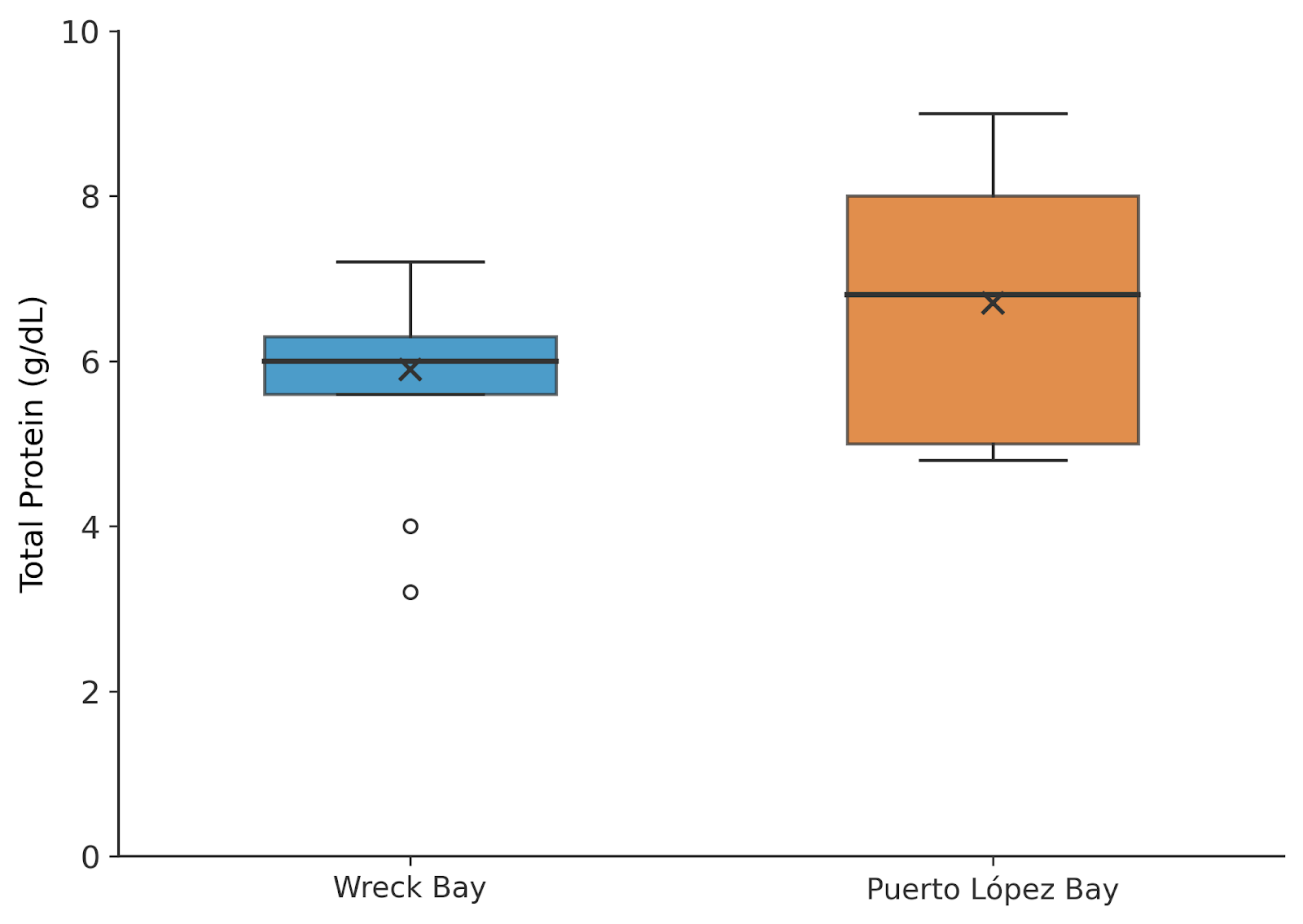
Total protein values comparison. Mean total protein concentrations in green sea turtles (*Chelonia mydas*) from mainland and insular sites. Total protein levels were slightly higher in turtles from Puerto López Bay (Mean = 6.70 g/dL) compared to those from Baquerizo Bay, San Cristóbal Island (Mean = 5.91 g/dL). However, this difference was not statistically significant (*p* = 0.16). Error bars represent standard deviation.

Biochemical analyses revealed significant differences between populations. Green turtles sampled in Puerto López Bay exhibited significantly higher blood glucose, BUN, and total protein levels compared to turtles sampled in Wreck Bay, Galápagos (Figs. 4–6). Mean blood glucose concentration in Puerto López Bay turtles was 122.6 mg/dL, compared to 69.2 mg/dL in Wreck Bay turtles (Fig. 4), with a statistically significant difference, *P* < 1 × 10^−5^. Mean BUN concentration was 75.6 in Puerto López Bay turtles and 18.87 in Wreck Bay turtles (Fig. 5), also showing a significant difference, *P* < 1  × 10^−4^. Mean total protein concentration was higher in turtles sampled from the mainland (6.70 g/dL) compared to turtles from Wreck Bay (5.91 g/dL; Fig. 6), although this difference was not statistically significant (*P* = 0.16).

All raw biochemical data and associated video files are available *via* the PeerJ data repository.

## Discussion

Hyperglycemia in sea turtles has been associated with physiological stress, hepatic or pancreatic disease, alterations in gluconeogenic pathways, and exogenous steroid exposure (Stacy & Innis, 2017a). Diet may also contribute to elevated glucose levels, although its role in green turtles remains incompletely understood. High-protein diets have been correlated with increased blood glucose concentrations in green turtles (Bloodgood et al., 2019). Renal function also influences glucose regulation, and elevated circulating glucose may result from reduced clearance or glucose loads exceeding renal excretory capacity. While stress can induce transient increases in blood glucose, this explanation is unlikely in the present study, as all turtles were captured and sampled using identical methods and appeared clinically healthy at the time of sampling. Although, chronic stress exposure from continuous anthropogenic stimulation cannot be ruled out at this time as a cause of elevated glucose levels. All turtles were assessed and marked as healthy using the following a modified visual veterinary external examination index (VEEI). The VEEI was adapted from Manire et al. (2017) and performed on each captured turtle. The mean index was calculated based on 11 external visual parameters rated on a scale of 1 (normal) to 2 (abnormal). The parameters assessed included: body condition (BC), mucus membrane (MM), hydration (H), pain score (PS), eye, ear, nose, and throat (EENT), skin (S), coelom (C), urogenital (U), musculoskeletal (MSK), and neuro (N), and whether or not there was any external or internal injury present.

Turtles exposed to greater levels of human activity in Puerto López Bay present increased blood urea nitrogen levels compared to the turtles from the less human-influenced environment. Blood urea nitrogen is a byproduct of the liver metabolizing protein that should be filtered and excreted *via* the kidneys as urea and ammonia (Salazar, 2014). In carnivorous species, BUN concentrations are typically higher, whereas in green turtles, BUN tends to become elevated with illness or increased protein intake (Stacy & Innis, 2017a). Although all the turtles in the study were within the normal established BUN levels, values from Puerto López Bay were consistently higher in the reference range than their counterparts in Wreck Bay (Aguirre & Balazs, 2000; Lewbart et al., 2014; Reséndiz & Lara-Uc, 2018; De Mello & Alvarez, 2019). It has been noted that sea turtles with increased protein intake often have increased BUN levels (Bloodgood et al., 2019). Since the turtles of Puerto López Bay have altered foraging habits compared to their counterparts in the Galápagos, the elevated BUN levels are likely related to diet and not primary disease. Green turtles’ BUN levels have been known to decrease after switching to a plant-based diet from a diet higher in protein (Bloodgood et al., 2019).

Total protein concentration is a multifactorial diagnostic indicator reflecting nutritional status, inflammation, infection, and protein metabolism in sea turtles (Stacy & Innis, 2017a). Elevated total protein values have been documented in populations that eat more meat and in younger juvenile green turtles that still have a diet primarily of animal protein (Bloodgood et al., 2019). Although turtles sampled in this study appeared in general good overall health, no further diagnostics or necropsies were performed to determine the presence of pathological issues and to fully evaluate potential subclinical disease processes.

Since the BUN and glucose values were determined by a point-of-care (POC) analyzer designed for mammals, the actual values might be slightly different when using a more standard, laboratory floor analyzer. Two papers compared the iSTAT and a sophisticated non-portable analyzer in various reptiles, including two tortoise species (McCain et al., 2010; Di Girolamo et al., 2018). They found a close correlation for some analytes and more varied results in others. Glucose values were close, especially at relatively low values (below 140 mg/dL). The reason we utilized the point-of-care iSTAT was primarily because of the portability, efficiency, and relatively low cost. Furthermore, we were working in remote locations without access to a clinical pathology laboratory. Finally, green turtles are a CITES 1 listed species, and legally we could not export blood samples to the USA without the proper permits. Neither the studies comparing BUN in two analyzers nor this study determined the total protein levels through whole blood centrifugation and a refractometer.

Green sea turtles foraging in heavily populated mainland areas and consuming fish and fish byproducts display elevated blood glucose and BUN levels compared to primarily herbivorous Galápagos individuals. Although the total protein levels were not statistically significant, it should be noted that the turtles of Puerto López Bay had an increased average total protein when compared to their herbivorous counterparts.

All measured health parameters fell within established reference intervals (EclinPath Cornell University College of Veterinary Medicine, 2013; Abbott Point of Care Inc., 2020; Carpenter & Harms, 2023), suggesting that the observed biochemical differences reflect variation in diet and habitat use rather than overt pathology. However, interpretation of these findings is limited by sample size, the short temporal scope of sampling (one month), and behavioral observations derived from a single camera deployment with a restricted field of view.

Moveover, the data collected from the camera footage is limited to a narrow viewing frame. Further studies could include a wide lens camera to better capture behavior or mounting multiple cameras on conspecifics in the area to gain a more cohesive idea of the behavior of the Puerto López Bay population. Further studies in these locations should include a larger sample size, various maturity levels of turtles combined with dietary analyses such as stable isotope or stomach content studies, which would further clarify the relationship between anthropogenic food sources and turtle health. This would aid in understanding if it’s a localized event or if green turtles in closer proximity to urban areas in mainland Ecuador present similar values. Given the ecological importance of green turtles, continued investigation into how human activities influence their foraging behavior and physiological condition will be essential for informing effective conservation and management strategies.

Long-term sampling and expanded dietary analyses would improve understanding of the relationship between foraging ecology and biochemical health indicators in these populations. Future studies incorporating stomach content analysis, stable isotope analysis, or fatty acid profiling, similar to work conducted in Gorgona National Park (Amorocho & Reina, 2007), would help clarify the extent to which anthropogenic food sources influence long-term physiological condition in green turtles.

## Conclusions

This study provides baseline data on selected biochemical markers in green turtles (*Chelonia mydas*) from both the Ecuadorian continental and the Galápagos National Park/Marine Reserve. While turtles from Puerto López Bay exhibited higher blood glucose and blood urea nitrogen compared to turtles from Wreck Bay, these values remained within established reference intervals and do not constitute a comprehensive assessment of health status.

This data report documents biochemical variation associated with contrasting foraging environments, including anthropogenic food sources, rather than diagnosing physiological impairment. Given the limited sample size, temporal scope, and restricted number of biomarkers analyzed, these findings should be interpreted as preliminary and descriptive. Expanded sampling across seasons, life stages, and additional physiological markers will be necessary to evaluate potential long-term implications of human-mediated foraging.

## Supplemental Information

10.7717/peerj.21367/supp-1 Supplemental Information 1 Raw data

10.7717/peerj.21367/supp-2 Supplemental Information 2 ARRIVE checklist

## Additional Information and Declarations

### Field Study Permissions

The following information was supplied relating to field study approvals (i.e., approving body and any reference numbers): The Galapagos National Park and the Machalilla National Park approved the study (permit No. PC- 32-21); permit No. MAE-EA-2021).

### Data Availability

The following information was supplied regarding data availability: The raw data is available in the Supplemental File.
